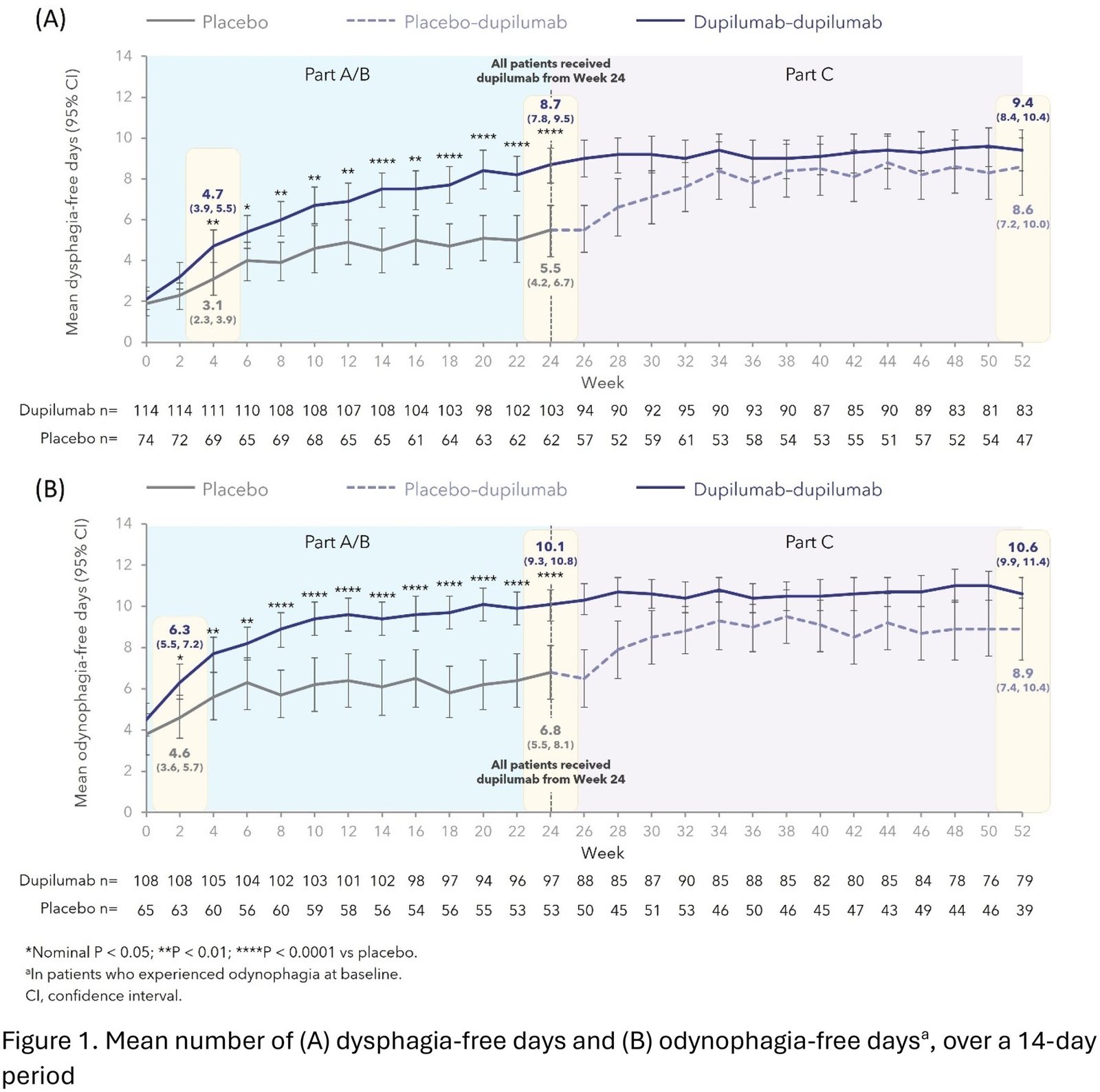# Poster Session II - A195 DUPILUMAB IMPROVES DYSPHAGIA, ODYNOPHAGIA, CHEST PAIN, AND HEARTBURN IN ADULT AND ADOLESCENT PATIENTS WITH EOSINOPHILIC ESOPHAGITIS UP TO 52 WEEKS: POST HOC ANALYSIS OF THE LIBERTY EOE TREET STUDY

**DOI:** 10.1093/jcag/gwaf042.195

**Published:** 2026-02-13

**Authors:** E Dellon, M Gupta, M Chehade, C Ma, F Racca, R A Pollock, C Gonzalez, C Cazeau, B Raphael, S T Tilton, R B Thomas

**Affiliations:** University of North Carolina School of Medicine, Chapel Hill, NC; University of Calgary, Calgary, AB, Canada; Icahn School of Medicine at Mount Sinai, New York, NY; University of Calgary, Calgary, AB, Canada; IRCCS Humanitas Research Hospital, Rozzano, Milan, Italy; Sanofi, Toronto, ON, Canada; Regeneron Pharmaceuticals Inc., Tarrytown, NY; Sanofi, Cambridge, MA; Regeneron Pharmaceuticals Inc., Tarrytown, NY; Sanofi, Cambridge, MA; Regeneron Pharmaceuticals Inc., Tarrytown, NY

## Abstract

**Background:**

Dysphagia, odynophagia, chest pain, and heartburn are symptoms that disrupt the daily lives of patients with eosinophilic esophagitis (EoE). Dupilumab, a fully human monoclonal antibody, is approved in the US and EU for EoE in patients aged ≥1 year weighing ≥15 kg. In the pivotal phase 3 LIBERTY EoE TREET study (NCT03633617), dupilumab 300 mg weekly (qw) significantly improved dysphagia vs placebo at Week (W)24.

**Aims:**

We assessed the impact of continued dupilumab use on dysphagia, odynophagia, chest pain, and heartburn to Week 52.

**Methods:**

Data were pooled from patients aged ≥12 years, treated with dupilumab qw or placebo for 24 weeks in Parts A and B of LIBERTY EoE TREET who continued to Part C and received dupilumab qw to W52. The number of days without dysphagia (Dysphagia Symptom Questionnaire [DSQ] question [Q] 2) and number of days without odynophagia (DSQ Q4; answered only if dysphagia is reported in Q2) over a 14-day period were analyzed at baseline and every 2 weeks to W52. Odynophagia outcomes were assessed in patients with ≥1 day of odynophagia in the 14 days prior to baseline. The proportion of patients without both chest pain and heartburn over the previous 7 days was measured by the EoE Symptom Questionnaire (EoE-SQ), which assesses 5 symptoms (chest pain, stomach pain, heartburn, regurgitation, throwing up) on a 5-point scale (1=never, 5=more than once a day) at W24 and W52.

**Results:**

At W24, the mean number of days without dysphagia and without odynophagia was greater in patients on dupilumab vs placebo at W24 (no dysphagia: 8.7 vs 5.5 days; no odynophagia: 10.1 vs 6.8 days respectively; both nominal *P*<0.0001) (**Fig. 1**). By W52, the mean number of days without dysphagia and without odynophagia increased in patients switching from placebo to dupilumab (no dysphagia: 8.6 days; no odynophagia: 8.9 days) and was maintained in patients continuing dupilumab (no dysphagia: 9.4 days; no odynophagia: 10.6 days) (**Fig. 1**). At W24, a greater proportion of patients on dupilumab had no chest pain or heartburn vs placebo (48.6% vs 28.4%, nominal *P*<0.01). By W52, the proportion of patients with no chest pain or heartburn increased in the group who switched from placebo to dupilumab at W24 (58.5%) and was maintained in patients continuing dupilumab (54.3%).

**Conclusions:**

Dupilumab improved dysphagia, odynophagia, chest pain, and heartburn vs placebo, at W24 in patients with EoE. Improvements were further maintained to W52.

**Funding Agencies:**

Sanofi and Regeneron Pharmaceuticals Inc.